# Trauma and Triage: Applying the Dick and Carey Instructional Design Model to a Primary Survey Clinical Workshop

**DOI:** 10.7759/cureus.8656

**Published:** 2020-06-16

**Authors:** Maxwell J Jabaay, Leah O Grcevich, Dario A Marotta, Joseph H Reynolds

**Affiliations:** 1 Department of Research, Alabama College of Osteopathic Medicine, Dothan, USA; 2 Department of Neurology, Division of Neuropsychology, University of Alabama, Birmingham, USA; 3 Department of Leadership and Professional Studies, Troy University, Montgomery, USA

**Keywords:** instructional design, primary survey, trauma, curriculum development and evaluation

## Abstract

Instructional design in the setting of medical education can be challenging. Multiple instructional design methods exist and have been documented in the literature. However, detailed applications of these models in the context of medical education are underreported. This technical report describes the application of a specific instructional design model to an acute care curriculum. Specifically, we illustrate the Dick and Carey instructional design model used at a one-day clinical workshop aimed at improving medical student exposure to the primary survey.

## Introduction

The primary survey (PS) is a rapid and systematic approach to evaluating critically ill or injured patients that focuses on airway, breathing, circulation, disability, and exposure (ABCDE) management [1]. This approach optimizes care team communication and efficiency while prioritizing threats to life and reducing adverse patient outcomes [2]. As such, the PS has become a core competency in undergraduate and graduate medical education [3]. Successfully performing a PS in a high-stress and rapidly evolving trauma situation is challenging for many students [4-5]. Efforts have been made to increase student exposure to trauma management in the clinical phase of medical education. However, the life-threatening nature of clinical encounters that require a primary survey has inherent risk. In many cases, the role of the primary surveyor is assumed by senior clinicians, further limiting student involvement and experience [6-7]. Thus, we set out to design a curriculum to help medical students gain proficiency and confidence in the primary survey using an instructional design technique known as the Dick and Carey model. 

The Dick and Carey model is considered one of the foremost Analysis, Design, Development, Implementation, and Evaluation (ADDIE) models, popular in industry, business, and academic environments. Since its first introduction in 1968, the model has been updated several times and is now described in detail in the 8th edition of Dick, Carey, and Carey’s *The Systematic Design of Instruction* [8]. The Dick and Carey model has been successfully applied to medical curricula in the past, thus we elected to use this model as the roadmap for our instructional design [9-10]. Realistic medical simulation has shown to improve medical student performance-based assessments in the management of simulated trauma; therefore, simulation served as the framework by which we delivered our curriculum [4, 11]. In this technical report, we document design considerations, learning objectives, and assessments for a reproducible preclinical trauma workshop known as Trauma and Triage. 

## Technical report

**Figure 1:**
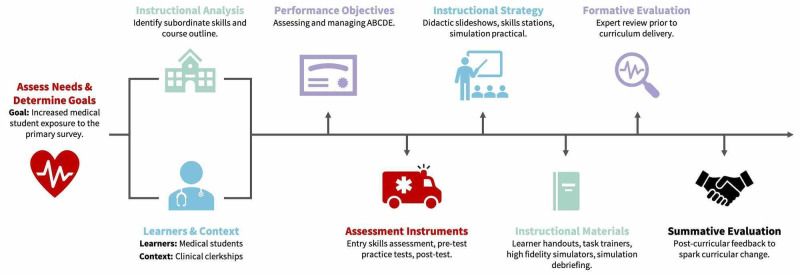
Steps of the Dick and Carey Instructional Model Adapted to a Primary Survey Curriculum Figure 1 depicts the steps of the Dick and Carey Model as it applies to a primary survey curriculum. The process starts with assessing the needs of institution and determining goals and progresses to the summative evaluation. It should be noted, continuous revisions should be made throughout the process.

This technical report illustrates the Dick and Carey model by applying it to a preclinical trauma curriculum [8]. Even with the development of seemingly similar curricula, it is important to apply these methods and adapt a curriculum based on specific learners and context. The Dick and Carey Model progresses through a series of steps and allows for continuous revision throughout the process. Figure 1 graphically represents the steps of the model.

Step 1: Assess needs and determine goals

The application of the Dick and Carey model begins by establishing whether or not a performance-based problem exists and whether or not the problem can be solved through additional education. Only then can the instructional design team continue with a *needs assessment*. A needs assessment identifies the desired performance level and compares it to current performance. This performance gap acts as a path to guide further curriculum development. We recommend reviewing the literature for existing curricula that may have addressed similar needs. This can streamline efforts and generate additional ideas to incorporate in your final design. Finally, it is important to develop instructional goals to help guide the design process.

For example, we identified an inherent gap in acute care management in a cohort of medical students. An informal interview revealed students felt they had learned technical skills, such as basic life support (BLS) and advanced cardiac life support (ACLS); however, they had yet to utilize the PS to determine whether these skills would apply in a rapidly evolving trauma situation. While this did not elucidate a quantitative performance deficit, it illustrated a working example of a gap in clinical knowledge that could benefit from instruction. A literature review revealed similar concerns at other medical institutions without evidence of established acute care curricula [4-5]. Following our needs assessment, we set out to achieve our instructional goal of creating a curriculum to improve preclinical exposure to the primary survey through education aimed at managing a patient’s airway, breathing, circulation, disability, and exposure.

Step 2: Analyze learners and context 

The next step has two distinct aspects: (1) analyzing the learners, and (2) considering the future environment where these skills would be performed (herein referred to as the context) [8]. For instance, we identified first- and second-year medical students with varying levels of simulation and medical experience as learners. These students desired to improve proficiency in the PS, a skill used frequently in the context of inpatient medical school rotations. 

Steps 3 and 4: Instructional analysis and performance objectives

The third and fourth steps of the Dick and Carey model involve an *instructional analysis* to design the layout of the curriculum in order to achieve the instructional goals [8]. The composition of the instructional analysis involves translating instructional goals into actionable performance objectives consisting of three unique components: *condition statement*, *behavior statement*, and a *criteria statement* [8]. 

A *condition statement* establishes the environment in which a scenario will take place. This gives the learner context in terms of preceding or triggering events and available resources. Condition statements can be manipulated by instructors to increase or decrease complexity based on the preceding analysis of learner experience [8]. 

The *behavior statement* describes the actions learners must take to achieve an objective [8, 12]. Behavior statements should be specific and actionable to avoid ambiguity. For instance, verbs that should be avoided include: "know," "understand," and "appreciate" because they lack specificity [8, 12]. 

A *criteria statement* sets performance expectations for how a learner’s performance will be graded or judged in future assessments.

**Figure 2:**
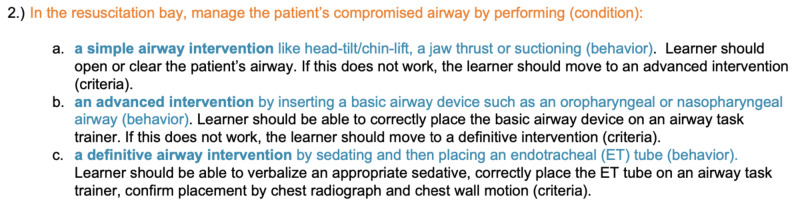
Example of a Performance Objective for Airway Management

A detailed set of performance objectives helps to streamline assessment creation and reduce tangential instructional material. To illustrate this, Figure 2 provides a color-coded example of one of the 10 performance objectives created for our instructional goal related to airway management. Conditional statements are colored in orange, behavioral statements are colored in blue, and criteria statements are colored in black. We recommend using this syntax for performance objective creation as it streamlines productivity and ensures each performance objective contains the proper components. A complete list of performance objectives used in the development of Trauma and Triage is provided in appendix 1.

**Figure 3:**
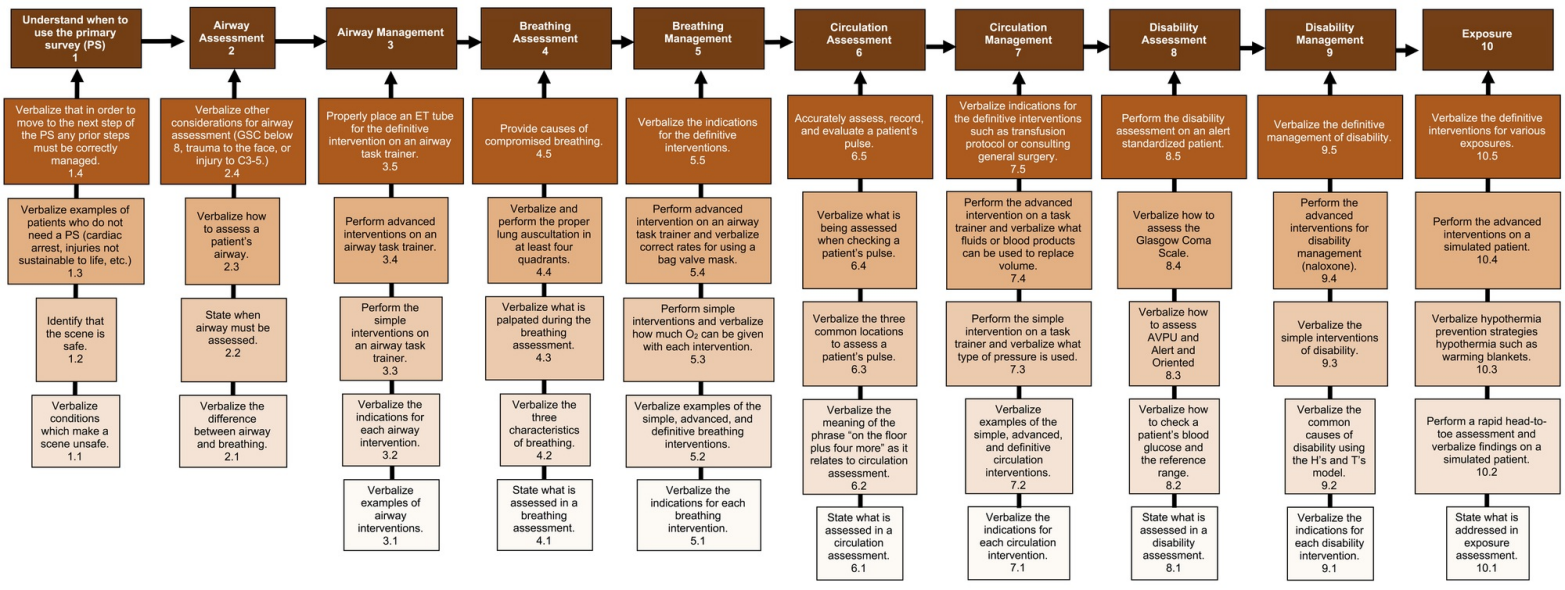
Subordinate Skill Flowchart for Primary Objectives Subordinate skill flow-charts must be made for each primary objective with more basic introductory skills at the bottom transitioning to more advanced skills above.

Finally, for each performance objective, subordinate or prerequisite skills must be identified. A subordinate skill is a foundational skill required for a learner to successfully achieve a performance objective. As an illustration, Figure 3 depicts examples of subordinate skill flow-charts for performance objectives of the Trauma and Triage curriculum. In the example of airway management, a subordinate tree begins with the most basic required skills (such as verbalizing the indications for airway intervention) and progresses to more advanced psychomotor skills (such as performing intubation on a task trainer).

Step 5: Assessment instruments

Once performance objectives are established, various assessments are required to evaluate whether or not a learner has accomplished a specific performance objective. These assessments include an *entry skills test*, *pre-test*, *practice tests, *and *post-tests* [8].

*Entry skills tests* are intended to screen learners for the foundational knowledge required to be successful in a particular program. Learners who do not possess a minimum competency should be referred for remediation prior to enrolling in the curriculum.

*Pre-tests* are pre-curricular assessments which gauge a group’s foundational proficiency with the educational material. Pre-tests are different from entry-skills tests in that they are not used to evaluate a minimum level of competency. Instead, they identify strengths and weaknesses in the group’s knowledge related to course material. In doing so, this allows instructors to tailor curriculum design toward a group of participants and maximize potential educational outcomes [8]. On a practical level, questions used to assess entry-skills are often combined with pre-test questions and evaluated differently based on answer selection.

*Practice tests* are intracurricular evaluations that provide learners with instant performance feedback for a particular skill or objective. For a learner, practice tests function as a learning tool. For instructors, practice tests identify content areas which could use additional reinforcement or clarification. In either situation, practice tests allow both instructors and learners an opportunity to correct performance before the final post-test evaluation.

*Post-tests* are the final evaluations used to determine whether performance objectives have been met. Post-tests should reflect the type of objective being evaluated. For instance, technical skills should be evaluated with practicums, while verbal skills should be evaluated with oral inquisitions. 

**Figure 4:**
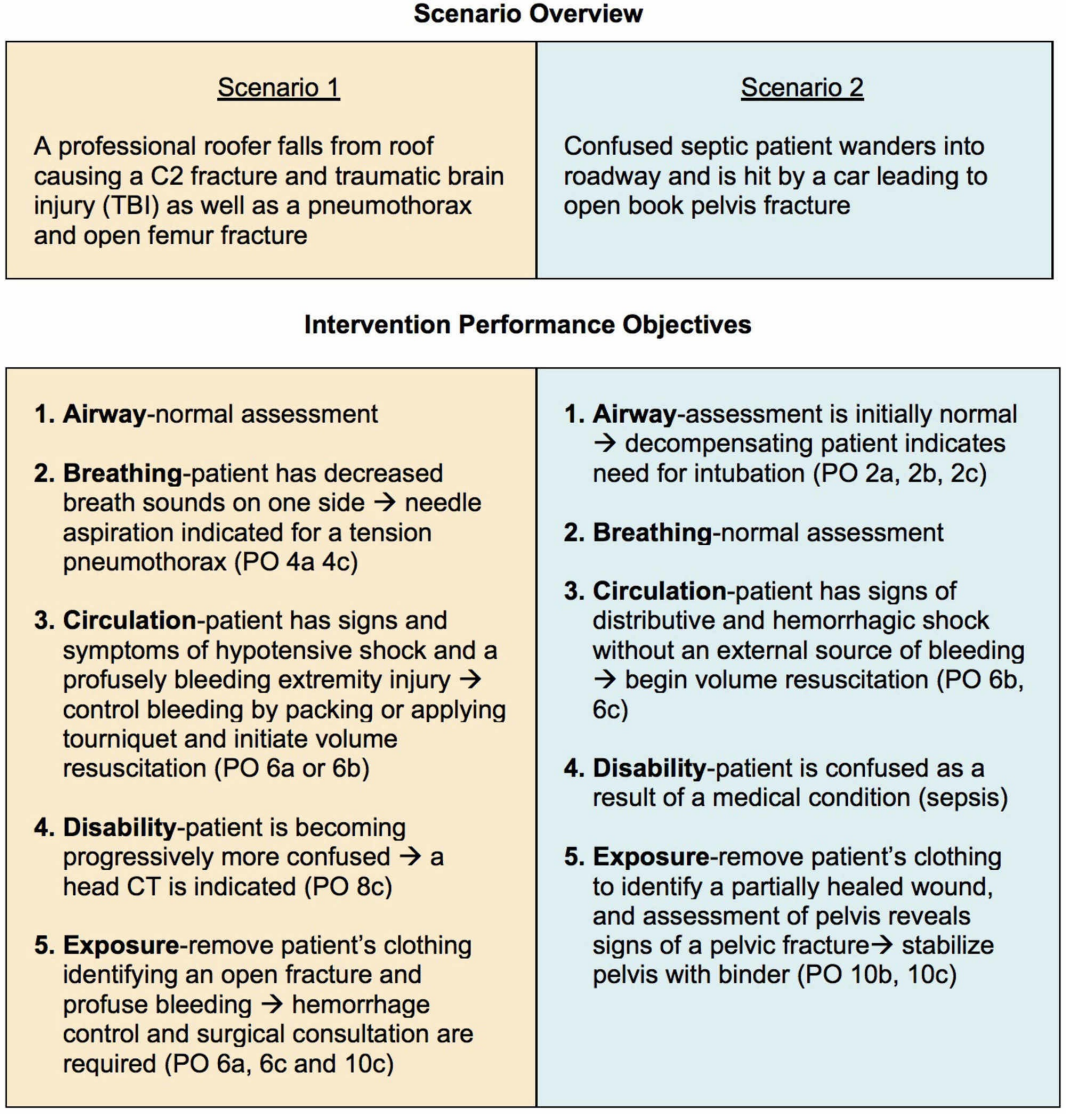
Simulation Scenarios Derived from POs Figure 4 references performance objectives (POs). Every case requires the performance of each assessment PO (PO 1, 3, 5, 7, 9), however, management steps (PO 2, 4, 6, 8, 10) are determined based on the specific findings in each simulation.

In the setting of Trauma and Triage, entry skills tests and pre-tests were combined into a single evaluation and are provided as a reference in appendix 2. Multiple choice questions and answers were used for the evaluation. Incorrect multiple-choice questions will be used to modify the group’s curriculum. Excessive incorrect responses flagged potential learners for remediation. Five practice-tests were developed to be used during the curriculum and are provided as a reference in appendix 3. Two simulated acute care scenarios were used as post-tests to demonstrate proficiency with the PS and are outlined in Figure 4. 

Step 6: Instructional strategy

After producing curricular evaluations, performance objectives are used to establish instructional strategies, which should align with teaching modalities. For instance, didactic modules can be used for objectives related to memorizing facts, while task-based learning should be used for application or psychomotor-related objectives. Once teaching modalities have been selected for each performance objective, logical considerations can be addressed, such as reserving a location, procuring equipment, and identifying qualified instructors. For example, Trauma and Triage utilized low fidelity task-trainers to teach methods of assessing patients during the PS, while didactic sessions were reserved for discussing potential causes of disability through gross pathology slides and radiographic imaging.

Step 7: Instructional materials

After developing an instructional strategy, the next step is to determine which instructional materials best suit the educational purpose. Instructional materials can include self-made slideshows, note packets, and worksheets. We recommend considering materials that balance short-term and long-term retention. For instance, while slideshows and interactive media are more engaging as a short-term learning tool, note packets and workbooks provide students with a lasting reference to use in the future. Trauma and Triage used a combination of materials including slideshow presentations, printed workbooks, and open-source literature [1]. Since simulations were used in the course, consolidated versions of the performance objectives were reviewed with participants following simulation exercises. A well-structured debrief is critical for student learning and has been shown to significantly improve learning outcomes compared to those who did not receive debriefing [13].

Step 8: Formative evaluation of instruction

The progress thus far constitutes a preliminary curriculum. An independent review by a subject matter expert, known as a *formative evaluation*, establishes the potential for the curriculum to accomplish the instructional goals. The formative evaluation serves to improve the effectiveness of the course [8]. In the case of Trauma and Triage, we consulted several board-certified emergency physicians and medical education experts. By doing so, we improved the quality of the course by applying feedback. Through this review, we also established that the instructional design had the potential to achieve our instructional goals.

Step 9: Summative evaluation

The final step of the Dick and Carey instructional design model calls for a *summative evaluation*. After the completion of the curriculum, it is necessary to solicit post-hoc feedback. This may come in the form of personal interviews, group interviews, or questionnaires. Surveys can be administered as written documents or in an on-line format. In the case of Trauma and Triage, we elected to conduct one-on-one personal interviews. Personal interviews gave us the latitude of expanding on learner feedback to further improve the curriculum.

## Discussion

The Analysis, Design, Development, Implementation, and Evaluation (ADDIE) model has been cited as an instructional design element in the medical education literature [10-11]. However, little detail has been provided on how these models were applied. Further, the literature lacks a detailed technical report documenting the application of such a model in the context of medical education. Thus, our goal was to mend this gap in the literature and provide medical educators with a framework with which to approach similar curricular designs. 

Instructional design can be an arduous and overwhelming process. Even a relatively short curriculum, such as Trauma and Triage, requires considerable planning and effort to successfully achieve educational objectives. The Dick and Carey instructional design model can be applied to a variety of goal-directed simulation curricula [10-11]. We recommend this model as it ensures that proper learning objectives have been identified, that assessment tools highlight those learning objectives, and that the curriculum and its outcomes are valuable for the learner. 

## Conclusions

The Dick and Carey instructional design model is a systematic approach to curriculum development. In this technical report, we document the application of the Dick and Carey model to a one-day primary survey workshop. Here we demonstrate the ease of applying the Dick and Carey model in the setting of medical education, thereby serving as a suitable method for designing a curriculum even for the most novice instructional designer. 

## Figures and Tables

**Figure 5:**
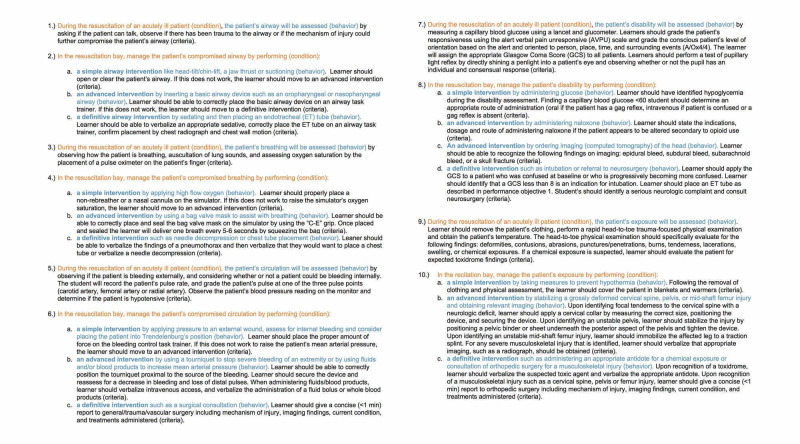
Primary Objectives for Primary Survey Curriculum

**Figure 6:**
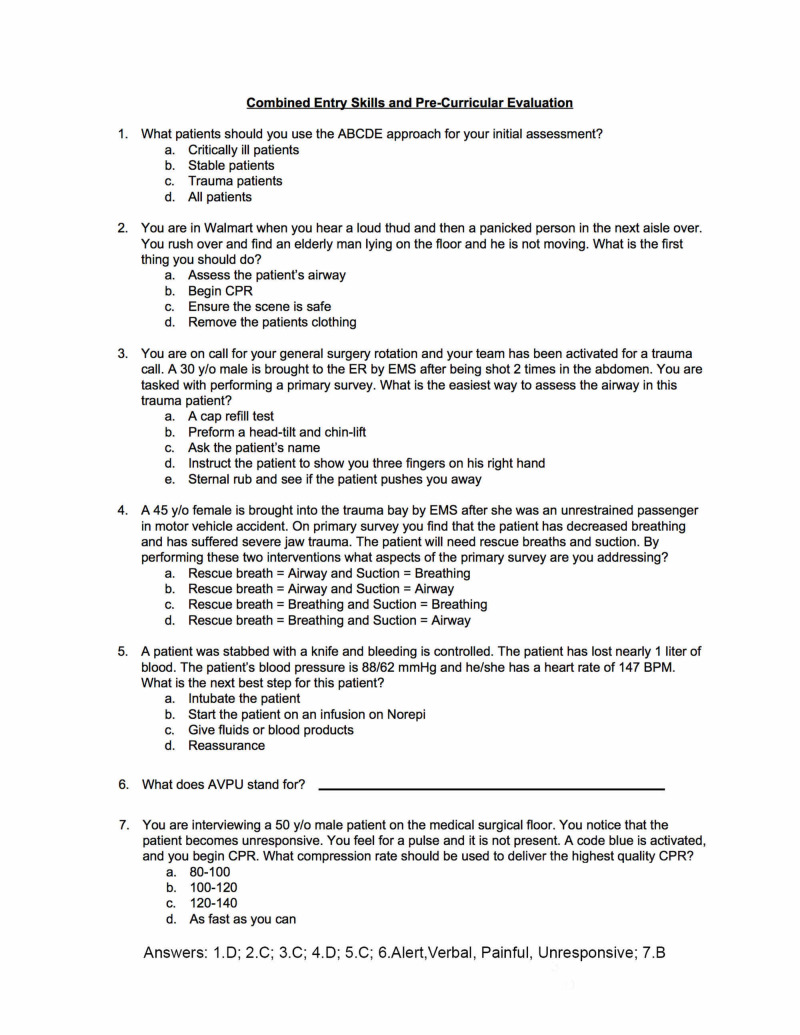
Entry Skills Test and Pre-Test Questions

**Figure 7:**
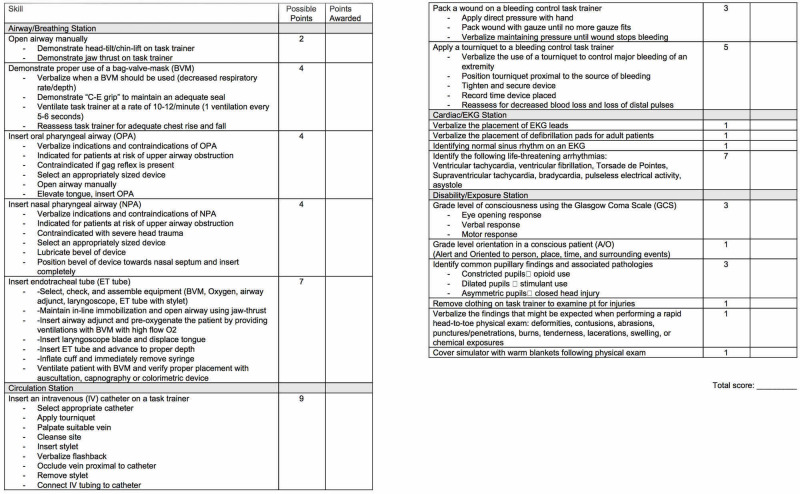
Practice Tests

## Appendices

Appendix 1

Appendix 2

Appendix 3 

## References

[1] Thim T, Krarup NH, Grove EL, Rohde CV, Løfgren B (2012). Initial assessment and treatment with the Airway, Breathing, Circulation, Disability, Exposure (ABCDE) approach. Int J Gen Med.

[2] Smith D, Bowden T (2017). Using the ABCDE approach to assess the deteriorating patient. Nurs Stand.

[3] Englander R, Flynn T, Call S (2016). Toward defining the foundation of the MD degree: core entrustable professional activities for entering residency. Acad Med.

[4] Ruesseler M, Weinlich M, Muller MP, Byhahn C, Marzi I, Walcher F (2010). Simulation training improves ability to manage medical emergencies. Emerg Med J.

[5] Gala PK, Osterhoudt K, Myers SR, Colella M, Donoghue A (2016). Performance in trauma resuscitation at an urban tertiary level I pediatric trauma center. Pediatr Emerg Care.

[6] Shaban S, Cevik AA, Canakci ME, Kuas C, El Zubeir M, Abu-Zidan F (2018). Do senior medical students meet recommended emergency medicine curricula requirements?. BMC Med Educ.

[7] Hammond J (2004). Simulation in critical care and trauma education and training. Curr Opin Crit Care.

[8] Dick W, Carey L, Carey JO (2015). The Systematic Design of Instruction. Pearson, Boston.

[9] Hsu TC, Lee-Hsieh J, Turton MA, Cheng SF (2014). Using the ADDIE model to develop online continuing education courses on caring for nurses in Taiwan. J Contin Educ Nurs.

[10] Reinbold S (2013). Using the ADDIE model in designing library instruction. Med Ref Serv Q.

[11] Ali J, Adam RU, Sammy I, Ali E, Williams JI (2007). The simulated Trauma Patient Teaching Module--does it improve student performance?. J Trauma.

[12] Gagné RM, Wager WW, Golas KC, Keller JM (2005). Principles of Instructional Design. Wadsworth/Thomson Learning, Belmont, CA.

[13] Savoldelli GL, Naik VN, Park J, Joo HS, Chow R, Hamstra SJ (2006). Value of debriefing during simulated crisis management: oral versus video-assisted oral feedback. Anesthesiology.

